# Caputo Fractional Derivative and Quantum-Like Coherence

**DOI:** 10.3390/e23020211

**Published:** 2021-02-09

**Authors:** Garland Culbreth, Mauro Bologna, Bruce J. West, Paolo Grigolini

**Affiliations:** 1 Center for Nonlinear Science, University of North Texas, P.O. Box 311427, Denton, TX 76201, USA; paolo.grigolini@unt.edu; 2 Departamento de Ingeniería Eléctrica-Electrónica, Universidad de Tarapacá, 1000000 Arica, Chile; mauroh69@gmail.com; 3 Army Research Office, DEVCOM US Army Research Laboratory, Research Triangle Park, NC 27708, USA; brucejwest213@gmail.com

**Keywords:** crucial events, fractional derivatives, complexity, cognition

## Abstract

We study two forms of anomalous diffusion, one equivalent to replacing the ordinary time derivative of the standard diffusion equation with the Caputo fractional derivative, and the other equivalent to replacing the time independent diffusion coefficient of the standard diffusion equation with a monotonic time dependence. We discuss the joint use of these prescriptions, with a phenomenological method and a theoretical projection method, leading to two apparently different diffusion equations. We prove that the two diffusion equations are equivalent and design a time series that corresponds to the anomalous diffusion equation proposed. We discuss these results in the framework of the growing interest in fractional derivatives and the emergence of cognition in nature. We conclude that the Caputo fractional derivative is a signature of the connection between cognition and self-organization, a form of cognition emergence different from the other source of anomalous diffusion, which is closely related to quantum coherence. We propose a criterion to detect the action of self-organization even in the presence of significant quantum coherence. We argue that statistical analysis of data using diffusion entropy should help the analysis of physiological processes hosting both forms of deviation from ordinary scaling.

## 1. Introduction

The origin of cognition is a subject of spirited debate among a number of research camps having significantly different views of the underlying mechanisms. Herein we restrict our discussion to two of the major contenders. One group consists of advocates of criticality, who interpret the dynamics of the brain as being the result of neuronal interactions that generate a transition from the condition of equilibrium statistical physics, with short-range correlation and simple scaling, to a non-equilibrium condition characterized by long-range correlations and scaling indices that often diverge [1]. The second camp of interest here consists of advocates of Quantum Probability Theory (QPT), who interpret the deviations from ordinary statistical physics as an indication that classical probability should be replaced by QPT [2,3,4]. The results of psychological experiments done by Tversky and Kahneman [5] lend support to the QPT advocates, who use quantum logic and QPT to interpret the psychological phenomenon of bounded rationality [6].

In this article we refer to criticality along the lines of the earlier work in [7]. We refer to this form of criticality as self-organized temporal criticality (SOTC), meaning that the process of transition to criticality is spontaneous and does not require fine tuning of an external control parameter. This criticality is called temporal because, adopting the view of turbulence, it generates short time regions of chaotic behavior punctuating large time regions of regular behavior, called laminar regions. The waiting-time probability density function (PDF) of the laminar regions is inverse power law (IPL) with an index μ ranging from 1 to 3. The work of Bohara et al. [7] establishes a bridge between the regular harmonic behavior and crucial events through a theoretical procedure called subordination to harmonic motion. We interpret the theoretical procedure as a combination of criticality generating crucial events along with coherence of quantum origin. We show that the work of [7], which is based on the analysis of brain dynamics data, may offer a bridge between the two apparently conflicting proposals as to the origin of cognition.

To understand the importance of this bridge we review the infinite memory of fractional Brownian motion (fBm), a theory without crucial events, and the continuous time random walk [8] (CTRW), a theory with crucial events and very well described by a fractional diffusion equation. We also stress the phenomenological attempt done in the recent past to establish a theoretical bridge between the theories of fBm and crucial events.

### 1.1. Fractional Brownian Motion

Fractional Brownian motion (fBm) [9] is compatible with equilibrium statistical mechanics. It is based on the rate equation for the dynamic variable X(t) describing diffusion:

(1) $$\dot{X}=\xi .$$

In fBm the stationary correlation function of the noise ξ(t), for ordinary diffusion having a very short correlation time $\tau_{c}$, is replaced by a correlation function $\Phi_{\xi }\left(\tau \right)$, which is still stationary but not integrable and has a diverging correlation time. The solution to Equation (1) relates the autocorrelation functions:

(2) $$\langle X\left(t_{1}\right)X\left(t_{2}\right)\rangle \equiv C\left(t_{1},t_{2}\right)\equiv \langle \xi^{2}\rangle \int_{0}^{t_{2}}dt_{2}^{\prime }\int_{0}^{t_{1}}dt_{1}^{\prime }\Phi_{\xi }\left(\left|t_{2}^{\prime }-t_{1}^{\prime }\right|\right),$$

where the normalized stationary autocorrelation is defined by

(3) $$\Phi_{\xi }\left(\left|t_{2}-t_{1}\right|\right)\equiv \frac{\langle \xi \left(t_{2}\right)\xi \left(t_{1}\right)\rangle }{\langle \xi^{2}\rangle }.$$

The proper choice of autocorrelation function, setting $t=t_{2}-t_{1}$, and sending *t*, $t_{1}$ and $t_{2}$ to *∞* reduces Equation (2) to the fBm proposed by Mandelbrot [10]:

(4) $$\frac{\langle X(-t)X(t)\rangle }{\langle X{\left(t\right)}^{2}\rangle }=1-2^{2H-1}.$$

Equation (2) yields:

(5) $$\langle X^{2}\left(t\right)\rangle =2\langle \xi^{2}\left(t\right)\rangle \int_{0}^{t}dt^{\prime }\int_{0}^{t^{\prime }}dt^{\prime \prime }\Phi_{\xi }\left(t^{\prime \prime }\right).$$

Assuming that:

(6) $$\langle X^{2}\left(t\right)\rangle \propto t^{2H},$$

and differentiating twice with respect to time, we obtain:

(7) $$\Phi_{\xi }\left(t\right)\propto \frac{1}{t^{\delta }}\mathrm{sign}\left(1-\delta \right),$$

establishing the relation between the IPL index δ and the scaling index δ=2−2H.

We have used *H* as adopted by Mandelbrot to denote the scaling first implemented by Hurst [11,12] (We note that the the authors of [12] pointed out that there exist anomalous forms of diffusion signaled by H≠0.5 departing from the Gaussian assumption of Mandelbrot. This is a consequence of confusion between two different forms of deviation from ordinary diffusion. The deviation due to crucial events may also deviate from the Gaussian assumption.). There exist alternative ways to generate scaling that deviates from the ordinary [8]. In the present article we denote the scaling with δ rather than *H*, and refer to this as a Fractional Diffusion Equation (FDE).

### 1.2. Fractional Diffusion Equation

The Montroll–Weiss (MW) perspective can be based on the interpretation that there are two distinct, but related, time scales associated with the dynamics of the system. First is the external time scale associated with an objective observer recording the behavior the of the system, called experimental or clock time. Second is the internal time associated with the internal dynamics of the system, called subjective or operational time. The experimental observation, done in the clock time *t*, is subordinated to a process occurring in the operational time *n*. We assume *n* to be an integer number large enough to become indistinguishable from continuous time. In operational time the diffusion equation is the ordinary diffusion equation:

(8) $$\frac{\partial }{\partial n}p\left(x,n\right)=D\frac{\partial^{2}}{\partial x^{2}}p\left(x,n\right).$$

In this representation ξ(t) of Equation (1) is completely random. However, in clock time the event ξ(n) occurs at time t(n) and the event ξ(n+1) at time t(n+1) with the time distance τ(n)=t(n+1)−t(n) derived from a waiting-time PDF ψ(τ). We are interested in the case where the waiting-time PDF has the hyperbolic form:

(9) $$\psi \left(\tau \right)=\left(\mu -1\right)\frac{T^{\mu -1}}{{\left(\tau +T\right)}^{\mu }}.$$

We use this hyperbolic PDF to define the temporal properties of crucial events. As stressed earlier, crucial events are defined by the time laminar regions, the time intervals separating the occurrence of consecutive events. This is a waiting-time PDF with the same asymptotic behavior in time as Equation (9), with the condition 1<μ<3. Herein we mainly study the case 1<μ<2. (In Section 2.3 we discuss the case 2<μ<3, where the time region between two consecutive crucial events is filled with either *W* or −W, according to random coin tossing.This is another case where crucial events, due to the aging process turning μ into μ−1, generate dynamics that may be confused with fBm.) In clock time we use the CTRW [8], yielding:

(10) $$p\left(x,t\right)=\sum_{n=0}^{\infty }\int_{0}^{t}dt^{\prime }\psi_{n}\left(t^{\prime }\right)\Psi \left(t-t^{\prime }\right)e^{L_{D}n}p\left(x,0\right),$$

where the diffusion operator is defined by $L_{D}\equiv D\frac{\partial^{2}}{\partial x^{2}}$, and $\psi_{n}\left(t^{\prime }\right)$ is the probability that *n* events have occurred and that the last event took place at time $t^{\prime }$. Note that the operator $L_{D}$ represents the diffusion process generated by the fluctuations ξ with the probability density:

(11) $$\Pi \left(\xi \right)=\frac{1}{2}\left(\delta (\xi -\sigma )+\delta (\xi +\sigma )\right),$$

generating the diffusion coefficient $D=\frac{\sigma^{2}}{2}$. For μ<2 CTRW generates the scaling $\delta =\frac{\mu -1}{2}$. Performing the Fourier-Laplace representation of Equation (10) turns the exponential operator $e^{L_{D}n}$ into $e^{=k^{2}Dn}$. In Section 5 to make DEA more efficient we use:

(12) $$\Pi \left(\xi \right)=\frac{1}{2}\delta \left(\xi -\sigma \right),$$

replacing $e^{-k^{2}Dn}$ with $e^{ik\sigma n}$, yielding the scaling δ=μ−1.

Note that the key work of [7] rests on 2<μ<3. We purposefully select μ<2. In fact, the condition 2<μ<3 is compatible with the crucial fluctuations becoming stationary in the long-time limit, a condition generating the misleading impression that the crucial fluctuations are compatible with fBm.

For the formula given by Equation (10) to hold with *n* going to *∞*, we must assume that for the random walker to travel the distance *X* in a time *t* a virtual infinitely large number of events may occur, thereby implying that the diffusion coefficient *D* is extremely weak. In the case μ<2 the mean waiting time 〈τ〉 diverges, which provides an additional reason for the experimental observation *t* to be extremely large.

It can be proven [13] that Equation (10) is equivalent to the integro-differential phase space equation:

(13) $$\frac{\partial }{\partial t}p\left(x,t\right)=D\int_{0}^{t}dt^{\prime }\Phi \left(t-t^{\prime }\right)\frac{\partial^{2}}{\partial x^{2}}p\left(x,t^{\prime }\right),$$

where Φ(t) is the MW memory kernel related to the waiting-time PDF and $\psi \left(\tau \right)=\psi_{n=1}\left(\tau \right)$. In the Laplace transform representation, where $\hat{f}\left(u\right)$ denotes the Laplace transform of f(t), this relation is:

(14) $$\hat{\Phi }\left(u\right)=\frac{u\hat{\psi }\left(u\right)}{1-\hat{\psi }\left(u\right)}.$$

In the case where the index for the hyperbolic PDF, asymptotically is the IPL index, is in the interval 1<μ<2, using Equation (14) it is shown [14] that:

(15) $$\hat{\Phi }\left(u\right)=u^{1-\alpha },$$

which when inserted into the Laplace transform of Equation (13) and the inverse Laplace transform taken, yields the fractional diffusion equation (FDE):

(16) $$\frac{\partial^{\alpha }}{\partial t^{\alpha }}p\left(x,t\right)=D\frac{\partial^{2}}{\partial x^{2}}p\left(x,t\right).$$

Here the fractional time derivative adopted is of the Caputo form [15] with α=μ−1<1.

To connect the present paper to the field of cognition and to that of fractional derivatives we stress two aspects of these results. First, that psychological arguments may be used to interpret the connection between operational time and clock time, as was done in [16]. The operational time is a subjective time in this psychological context with a logarithmic connection with the clock time *t*, which changes an exponential waiting-time PDF into the hyperbolic structure of Equation (9). This property is why we consider the CTRW formalism to be closely connected to cognition. The earlier work of [17] analyzed a series of events with the waiting-time PDF in Equation (9) using the concept of Kolmogorov-Sinai complexity and found that the signal becomes computationally compressible for μ<3. This is equivalent to determining that the time series hosts messages that can be decoded. On the other hand, the Kolmogorov-Sinai entropy vanishes for μ<2, which has recently been generalized to take into account the rare crucial events [18] in this region. These crucial events are conjectured to be a signal of swarm intelligence [19], and observations brain dynamics indicate that μ=2 is a proper signature of the brain of an awake subject [20]. The events characterized by the inter-event or waiting-time PDF of Equation (9) are considered to be a signature of cognition and may be be responsible for the efficient transport of information from one intelligent system to another [21]. The term *crucial events* acknowledges the importance of these rare events.

Second, concerning the fractional calculus, we note that the Caputo fractional derivative in Equation (8) makes this FDE equivalent to the anomalous diffusion equation with the time convolution of Equation (13). This implies deviation from the Markov condition, which may be confused with fBm memory. The infinite fBm memory does not have the effect of replacing the ordinary time derivative in Equation (8) with the Caputo fractional derivative [22]. Rather, it has the effect of turning the constant diffusion coefficient *D* into a time dependent diffusion coefficient $Dt^{2H-1}$. In this article we re-derive this result using the quantum formalism of Mori [23] and Weiss [24], advancing the phenomenological arguments in [22] with rigorous theoretical foundation to establish a bridge between crucial events and fBm. This might be interpreted as a bridge between classical probability, generating anomalous diffusion, and the QPT of [2,3,4,6]. However, we use the term “quantum-like coherence” rather than quantum coherence. We note also that Turalska and West [25] used a model of interacting units to mimic a decision making system and found that the behavior of the single units is very well described by Equation (8). This implies that the Caputo fractional derivative may have an important role to play in understanding the conjunction fallacy, that being, given two events A and B the probability P(A) is always greater than the conjoined probability P(A∪B).

### 1.3. Bridge between fBm and Crucial Events

We use quantum mechanical arguments to prove that the fBm yields a diffusion equation with a time-dependent diffusion coefficient:

(17) $$\frac{\partial }{\partial t}p\left(x,t\right)=Dt^{2H-1}\frac{\partial^{2}}{\partial x^{2}}p\left(x,t\right).$$

Bologna et al. [22] used fBm and CTRW jointly to study the behavior of a FDE with a time-dependent diffusion coefficient:

(18) $$\frac{\partial^{\alpha }}{\partial t^{\alpha }}p\left(x,t\right)=Dt^{2H-1}\frac{\partial^{2}}{\partial x^{2}}p\left(x,t\right),$$

and established that it leads to the relation between the scaling indices:

(19) $$\delta =\frac{2H-1+\alpha }{2}.$$

Recall that δ is the scaling index of the second moment.

### 1.4. Goal of This Paper

Equation (18) is a bridge between quantum coherence and crucial events. However, the derivation of [22] is phenomenological. An important goal of this paper is to establish its rigorous dynamical foundation. After establishing this bridge we use a special method of entropic analysis based on the earlier work of reference [26] to filter the fBm component. In the concluding remarks we discuss the benefits that the results of this paper are expected to offer in the debate on the origin of bounded rationality.

## 2. Memory of a System in Thermodynamical Equilibrium

A system consisting of a phase space variable *x* in contact with a heat reservoir schematically described by the set of variables $\left\{\xi ,z\right\}$ has dynamics that are described by the Liouville equation:

(20) $$\frac{\partial \rho (x,\xi ,z,t)}{\partial t}=\left(L_{0}+L_{1}\right)\rho \left(x,\xi ,z,t\right).$$

where ρ(x,ξ,z,t) is the phase space distribution function. The Liouville operator has two contributions. The Liouvillian $L_{0}$ refers to the set of reservoir variables $\left\{\xi ,z\right\}$, whereas $L_{1}$ couples the reservoir to the variable of interest so as to generate a diffusive process:

(21) $$L_{1}=-\xi \frac{\partial }{\partial x}.$$

The phase space variables are time independent and denoted by lower case letters.

The stochastic nature of the complex variable ξ is determined by its interaction with the set of variables z. We adopt the interaction picture in which the new phase space distribution function is defined:

(22) $$\tilde{\rho }\left(x,\xi ,z,t\right)=e^{-L_{0}t}\rho \left(x,\xi ,z,t\right),$$

which enables us to rewrite Equation (20) as the simpler looking equation:

(23) $$\frac{\partial \tilde{\rho }\left(x,\xi ,z,t\right)}{\partial t}={\tilde{L}}_{1}\left(t\right)\tilde{\rho }\left(x,\xi ,z,t\right),$$

where the coupling Liouvillian is given by:

(24) $${\tilde{L}}_{1}\left(t\right)\equiv e^{-L_{0}t}L_{1}e^{L_{0}t}.$$

Introducing the time-ordered exponential the solution of Equation (23) is:

(25) $$\tilde{\rho }\left(x,\xi ,z,t\right)=\overset{\leftarrow }{exp}\left\{\int_{0}^{t}dt^{\prime }{\tilde{L}}_{1}\left(t^{\prime }\right)\right\}\tilde{\rho }\left(x,\xi ,z,t=0\right).$$

Equation (25) can be simplified by assuming the factored equilibrium initial condition:

(26) $$\rho \left(x,\xi ,z,t=0\right)=p\left(x,t=0\right)\rho_{eq}\left(\xi ,z\right)\equiv p_{0}\left(x\right)\rho_{eq}\left(\xi ,z\right).$$

with a change in notation and recalling the property of the equilibrium reservoir distribution:

(27) $$L_{0}\rho_{eq}\left(\xi ,z\right)=0,$$

entailing the relation $e^{-L_{0}t}\rho_{eq}\left(\xi ,z\right)=\rho_{eq}\left(\xi ,z\right)$, so that

(28) $$\tilde{\rho }\left(x,\xi ,z,t\right)=\overset{\leftarrow }{exp}\left\{\int_{0}^{t}dt^{\prime }{\tilde{L}}_{1}\left(t^{\prime }\right)\right\}p_{0}\left(x\right)\rho_{eq}\left(\xi ,z\right).$$

We also make the assumption that the reservoir {ξ,z} is ideal; that in spite of its interaction with *x*, it does not depart from its equilibrium distribution density. Thus, from Equation (28) we obtain the equation of interest:

(29) $$\rho \left(x,\xi ,z;t\right)=e^{L_{0}t}\overset{\leftarrow }{exp}\left\{\int_{0}^{t}dt^{\prime }{\tilde{L}}_{1}\left(t^{\prime }\right)\right\}p_{0}\left(x\right)\rho_{eq}\left(\xi ,z\right),$$

which, due to the approximations made, can be projected into the functional form:

(30) $$\begin{array}{cc} p\left(x,t\right)\rho_{eq}\left(\xi ,z\right) & =P\rho (x,\xi ,z,t)\\ \; & =P\left(e^{L_{0}t}\overset{\leftarrow }{exp}\left\{\int_{0}^{t}dt^{\prime }L_{1}\left(t^{\prime }\right)\right\}p_{0}\left(x\right)\rho_{eq}\left(\xi ,z\right)\right). \end{array}$$

The factored form is a consequence of the projection operator being defined as a trace over the reservoir equilibrium distribution:

(31) $$P\left\{\cdot \right\}\equiv \rho_{eq}\left(\xi ,z\right)Tr_{E}\left\{\cdot \right\},$$

and the reduced PDF p(x,t) being defined by:

(32) $$p\left(x,t\right)\equiv Tr_{E}\left\{\rho (x,\xi ,z,t)\right\}.$$

Notice that the reservoir equilibrium distribution density must be normalized:

(33) $$Tr_{E}\left\{\rho_{eq}\left(\xi ,z\right)\right\}=1,$$

so that the projection operator is idempotent, $P^{2}=P$. and Equation (30) gives the explicit expression for the reduced PDF:

(34) $$p\left(x,t\right)=Tr_{E}\left\{e^{L_{0}t}\overset{\leftarrow }{exp}\left\{\int_{0}^{t}dt^{\prime }L_{1}\left(t^{\prime }\right)\right\}p_{0}\left(x\right)\rho_{eq}\left(\xi ,z\right)\right\}.$$

Note that the tracing done on Equation (33) has the effect of turning $e^{-L_{D}t}$ into 1.

When we apply the trace over the equilibrium distribution the operator $e^{L_{0}t}$ becomes unity using Equation (27). The trace is an integration over {ξ,z}, while $L_{0}$ is a differential operator over the same variables and consequently the phase space variable ξ is related to its dynamical counterpart by:

(35) $$\tilde{\xi }=e^{-L_{o}t}\xi e^{L_{o}t}=\xi \left(t\right).$$

Thus, the dynamic variable can be expressed in operator form as:

(36) $$\xi \left(t\right)\equiv \xi e^{L_{o}t}.$$

Taking into account that the reservoir equilibrium distribution is properly normalized, after some algebra, Equation (34) reduces to:

(37) $$p\left(x,t\right)=\langle \overset{\leftarrow }{exp}\left\{\int_{0}^{t}dt^{\prime }{\tilde{L}}_{1}\left(t^{\prime }\right)\right\}\rangle p_{0}\left(x\right),$$

which is true in general when the operator does not commute with itself at different times:

(38) $$\left[\tilde{L_{1}}\left(t_{1}\right),\tilde{L_{1}}\left(t_{2}\right)\right]\neq 0\;\mathrm{if}\;t_{1}\neq t_{2}.$$

We are using:

(39) $$\langle \xi \left(t_{1}\right)\xi \left(t_{2}\right)\rangle =\langle \xi e^{L_{0}\left(t_{1}-t_{2}\right)}\xi \rangle =\langle \xi_{eq}^{2}\rangle \Phi_{\xi }\left(\right|t_{1}-t_{2}\left|\right),$$

which is a stationary correlation function satisfying the condition:

(40) $$\langle \xi \left(t_{1}\right)\xi \left(t_{2}\right)\rangle =\langle \xi \left(t_{2}\right)\xi \left(t_{1}\right)\rangle ,$$

which allows us to interpret the fluctuation, ξ(t) as compatible with $[\xi_{1}\left(t\right),\xi_{2}\left(t\right)]=0$. This is a consequence of our approach to fBm. The property of Equation (37) is a result of the fact that quantum mechanics, as classican mechanics, is invariant under time reversal. To get fBm we make the Gaussian assumption, converting correlation functions of ξ with an arbitrary even number of times due to the assumption that these fluctuations are symmetric, into a product of two time correlation functions. Another way of obtaining this result is based on the observation that the adoption of the Wigner pseudo-probability [27,28] generates a Liouville-like equation with the Liouville operator being the sum of an operator identical to the classical operator and a term generating quantum effects, including quantum tunneling. However, the second term vanishes if the potential is linear. For this reason, although our derivation of fBm rests on quantum mechanics, we generate a form of coherence that is compatible with classical mechanics. This is the reason why we use the expression “quantum-like coherence” rather than quantum coherence to describe this part of the bridge.

We are using the property:

(41) $$\langle \left[\tilde{L_{1}}\left(t_{1}\right),\tilde{L_{1}}\left(t_{2}\right)\right]\rangle =0,$$

which is identical to Equation (49) in Yearsley and Busemeyer [6]. That study focused on the lack of commutation, P(A|B)≠P(B|A), where *A* and *B* refer to distinct decision-making processes. Here Equation (41) enables us to drop the time ordering in Equation (37) to obtain:

(42) $$p\left(x,t|x_{0}\right)=\langle e^{-\int_{0}^{t}dt^{\prime }\xi \left(t^{\prime }\right)\frac{\partial }{\partial x}}\rangle p_{0}\left(x\right).$$

In the case where Y(t) has Gaussian statistics we know [29] that:

(43) $$\langle e^{Y(t)}\rangle =e^{\frac{1}{2}\langle Y^{2}\left(t\right)\rangle }.$$

Setting the random operator to that of the interation Liouvillian:

(44) $$Y\left(t\right)\equiv -\int_{0}^{t}dt^{\prime }\xi \left(t^{\prime }\right)\frac{\partial }{\partial x},$$

and using Equation (43) we obtain:

(45) $$p\left(x,t\right)=e^{\frac{1}{2}\langle {\left(\int_{0}^{t}dt^{\prime }\xi \left(t^{\prime }\right)\right)}^{2}\rangle \frac{\partial^{2}}{\partial x^{2}}}p_{0}\left(x\right).$$

The average in Equation (45) is evaluated using the assumption that stochastic variable ξ(t) is Gaussian, stationary and with the IPL correlation function:

(46) $$\Phi_{\xi }\left(t\right)\propto sign\left(1-\eta \right)\frac{1}{t^{\eta }},$$

with the IPL index given by:

(47) η=2−2H,

which generates the connection with the second moment of the diffusion variable X(t):

(48) $$\langle {\left(\int_{0}^{t}dt^{\prime }\xi \left(t^{\prime }\right)\right)}^{2}\rangle ={\langle \xi^{2}\rangle }_{eq}\int_{0}^{t}dt_{1}\int_{0}^{t_{1}}dt_{2}\Phi_{\xi }\left(\right|t_{1}-t_{2}\left|\right)=2\langle X^{2}\left(t\right)\rangle .$$

Using the scaling property of the fluctuating velocity establishes the connection to fBm:

(49) $$\langle X^{2}\left(t\right)\rangle \propto t^{2H}.$$

The important result of this analysis is that the Liouville equation given by Equation (20) is, by the application of the operator P, projected from the schematic equation:

(50) $$\frac{\partial }{\partial t}\rho =\left(L_{0}-\xi \frac{\partial }{\partial x}\right)\rho ,$$

into the diffusion equation with a time-dependent diffusion coefficient:

(51) $$\frac{\partial }{\partial t}p\left(x,t\right)=Dt^{2H-1}\frac{\partial^{2}}{\partial x^{2}}p\left(x,t\right),$$

in agreement with Equation (17). In the diffusion case the PDF p(x,t) is always a conditional probability, implying that at t=0 the diffusional variable X(t) starts from the vanishing value X=0. Quantum coherence induced fBm, as we subequently show, makes this conditional probability deviate from the Markov condition. The Liouville-like formalism adopted to get Equation (51) is classical, but according to Wigner theory the linear case leads to results indistinguishable from those generated by the quantum formalism. For this reason we interpret Equation (51) to be a signature of a form of quantum-like coherence.

### 2.1. Mori Memory

Deviation from ordinary diffusion can be realized by changing the time-independent diffusion coefficient *D* into the time-dependent diffusion coefficient $Dt^{2H-1}$. This is a manifestation of the Mori memory [23] at the level of diffusion. The time convoluted structure of a generic quantum mechanical variable |A〉 is derived from its Heisenberg representation [30]:

(52) $$\frac{d}{dt}|A\left(t\right)\rangle =\Gamma |A\left(t\right)\rangle .$$

As a consequence of this equation of motion, the variable of interest is no longer confined to the original state |A〉=|A(0)〉 and it explores the entire Hilbert space of the operator Γ. Mori builds up a complete basis set moving from the state $|f_{0}\rangle =|A\rangle$ so as to obtain a tri-diagonal representation of the matrix Γ. Thus, without any approximation Equation (52) can be expressed in the time convoluted form:

(53) $$\frac{d}{dt}|A\left(t\right)\rangle =-k\int_{0}^{t}dt^{\prime }\Phi_{1}\left(t^{\prime }\right)|A\left(t-t^{\prime }\right)\rangle +|f_{1}\left(t\right)\rangle .$$

where the memory kernel $\Phi_{1}\left(t\right)$ is proportional to $\langle f_{1}|f_{1}\left(t\right)\rangle$. In the case where the quantum variable |A(t)〉 is related to the classical variable ξ(t), this equation corresponds to the stochastic differential equation:

(54) $$\frac{d}{dt}\xi \left(t\right)=-k\int_{0}^{t}dt^{\prime }\Phi_{\eta }\left(t^{\prime }\right)\xi \left(t-t^{\prime }\right)+\eta \left(t\right),$$

in which case the memory kernel $\Phi_{\eta }\left(t\right)$ is the stochastic representation of the autocorrelation function using the random variable η(t).

In the literature Equation (54) is referred to as Generalized Langevin Equation (GLE) because, in the case when η(t) is white noise, $\Phi_{\eta }\left(t\right)$ becomes equivalent to the delta function of Dirac and Equation (54) becomes identical to the standard Langevin equation. In the literature there has been little attention given to the transition from the quantum to the classical condition of GLE. The reason for this may be due to the nature of the Mori derivation of the time convoluted form of Equation (53). This theoretical treatment is based on the linear assumption, which is equivalent to assuming that the system under study is a set of oscillators interacting through harmonic couplings. The adoption of the formalism of the Wigner distribution shows that in this case the time evolution of the quantum Wigner pseudo-probability is indistinguishable from the time evolution of the classical phase space distribution [27]. Thanks to the central limit theorem, as pointed out by Tegmark and Shapiro [31], the behavior of infinite systems of coupled harmonic oscillators is characterized by Gaussian fluctuations, which affords strong theoretical support to the foundation of fBm, a Gaussian process, through the GLE. Following this line of reasoning Tegmark reached the conclusion that the brain is classical [32] and that it is unlikely that the brain is a computer [33]. He found that the decoherence time scales are of the order of $10^{-13}$ s, $10^{-20}$ s, very short compared the neuronal dynamics operating on relevant time scales of $10^{-3}$ s, $10^{-1}$ s.

Tegmark and Shapiro also afford an extension of the concept of a generalized coherent state [31]. Zurek and co-workers had shown that that coherent states are more robust than non-coherent states to the effects of their environment [34]. Arguing along the same lines, Tegmark and Shapiro reached the conclusion that nature is indeed filled with coherent states. One intuitive interpretation of this result is that a system of linearly interacting quantum oscillators is not affected by decoherence in-so-far as it corresponds to a condition of total equivalence between quantum and classical physics, when the pseudo-probability distribution of Wigner is adopted. On the other hand, quantum coherence underlying fBm may be the reason why, in spite of decoherence making the brain classical, the concept of consciousness as a state of matter [35] may benefit from the adoption of quantum logic.

### 2.2. Weiss Quantum Dissipation

In Mori’s rigorous quantum mechanical theory the scalar product $\langle A|A\left(t\right)\rangle =\langle f_{0}|f_{0}\left(t\right)\rangle$ is proportional to the correlation function $\Phi_{0}\left(t\right)=\Phi_{\xi }\left(t\right)$. In other words, the quantum-like decision making process rests on the integration over the variable $\xi \left(t\right)\propto \Phi_{\xi }\left(t\right)$, which can be described using the Weiss formalism [24] by a spectral decomposition:

(55) $$\Phi_{\xi }\left(t\right)=\int d\omega \rho \left(\omega \right)cos\left(\omega t\right).$$

This procedure is equivalent [13] to the adoption of the Onsager principle, making the regression to equilibrium of the fluctuations ξ(t) identical to the corresponding stationary correlation function $\Phi_{\xi }\left(t\right)$. The infinitely aged correlation function of Equation (55) is due to the superposition of regular oscillations with a spectral density of frequencies given by:

(56) $$\rho \left(\omega \right)\propto \omega^{\eta -1},$$

yielding Equation (46) with the IPL index η in the range 0 ≤η≤ 2 [24,36]. The IPL index of the frequency spectrum is related to scaling index for the diffusion time series *H* by:

(57) $$H=1-\frac{\eta }{2}.$$

When η<1 the correlation function $\Phi_{\xi }\left(t\right)$ has a slowly decaying non-integrable tail. When 1<η<2, the negative tail is integrable, but must exactly compensate for the positive portion of the normalized correlation function, so as to realize the condition:

(58) $$\int_{0}^{\infty }\Phi_{\xi }\left(t\right)dt=0.$$

This is equivalent to stating that under these conditions the very short correlation time $\tau_{c}$ of the totally random noise is turned into $\tau_{c}=\infty$.

The Onsager principle corresponds to replacing the mean value 〈ξ(t)〉 with the corresponding equilibrium autocorrelation function $\Phi_{\xi }\left(t\right)$. Here we make the assumption that 〈ξ(t)〉 is replaced by a classical value ξ(t) satisfying the condition that its autocorrelation function is $\Phi_{\xi }\left(t\right)$. Cakir et al. [36] used an algorithm that creates a time series {ξ(t)} generating that autocorrelation function. Although for the sake of computational simplicity we adopt the algorithm of Mandelbrot and van Ness [10], we relate the infinite memory of the fBm of Equation (4) to the quantum coherence of the Weiss formalism of Equation (55).

### 2.3. Quantum Coherence Deviates from the Markov Condition

The characteristic function is defined as the Fourier transform of the PDF, which has the formal expression:

(59) $$\langle e^{ikx}\rangle =\hat{p}\left(k,t\right).$$

In the case of an fBm process, using Equation (45) this is shown to be:

(60) $$\hat{p}\left(k,t\right)=e^{-k^{2}t^{2H}}.$$

As a consequence of H≠0.5 the characteristic function departs from the chain condition:

(61) $$\hat{p}\left(k,t_{2}\right)=\hat{p}\left(k,t_{1}\right)\hat{p}\left(k,t_{2}-t_{1}\right),$$

thereby violating the Markov property, which generates not only ordinary diffusion, but also Lévy scaling [37,38]. When the variable ξ is dichotomous, holding for instance the values *W* and −W, rather than Gaussian, as required to generate fBm, the time convoluted structure of Equation (13) is recovered. Allegrini et al. [38] studied the case where the variable ξ holds the values *W* and −W with the waiting-time PDF of Equation (9) and 2<μ<3. In this case in the long-time limit the memory kernel Φ(t) of Equation (13) is identical to the correlation function $\Phi_{\xi }\left(t\right)$ of Equation (46) with η<1. In this case however, the correlation function becomes stationary in the long-time limit as a result of an extended aging process due to crucial events turning μ into μ−1 and η=μ−1.

In full agreement with the observation that the condition of Equation (61) is a generator of both standard and Lévy diffusion, the authors of [38] proved that in this case the time convolution diffusion structure of Equation (13) becomes equivalent to the fractional diffusion structure $D\frac{\partial^{\beta }}{\partial {\left|x\right|}^{\beta }}p\left(x,t\right)$, where β=μ−1. The use of subordination in this case yields:

(62) $$\frac{\partial^{\alpha }}{\partial t^{\alpha }}p\left(x,t\right)=D\frac{\partial^{\beta }}{\partial {\left|x\right|}^{\beta }}p\left(x,t\right),$$

which is known to yield the scaling δ=α/β [39]. This interesting property is outside the scope of this paper, but we mention it to prevent possible confusion with ordinary fBm. Equation (62) is driven only by crucial events and its scaling does not have any connection with the infinite memory of fBm.

## 3. Activation of Crucial Events

To activate crucial events before applying the projection technique responsible for the emergence of quantum coherence we use the following equation:

(63) $$\frac{\partial^{\alpha }}{\partial t^{\alpha }}\rho \left(x,\xi ,z,t\right)=\left(L_{0}+L_{1}\right)\rho \left(x,\xi ,z,t\right).$$

According to Pramukkul et al. [14] this fractional differential equation is equivalent to the integro-differential equation:

(64) $$\frac{\partial }{\partial t}\rho \left(x,\xi ,z,t\right)=\int_{0}^{t}dt^{\prime }\Phi \left(t^{\prime }\right)\left(L_{0}+L_{1}\right)\rho \left(x,\xi ,z,t-t^{\prime }\right).$$

Using the arguments that led us to represent Equation (20) in the more convenient form of Equation (50), we rewrite Equation (63) in the form:

(65) $$\frac{\partial }{\partial t}\rho \left(x,\xi ,z,t\right)=\int_{0}^{t}dt^{\prime }\Phi \left(t-t^{\prime }\right)\left(L_{0}-\xi \frac{\partial }{\partial x}\right)\rho \left(x,\xi ,z,t^{\prime }\right).$$

We then apply the psychological interpretation to the global system, the system of interest plus its environment. We interpret the fluctuations of the global system as being perceived on a psychological time scale connected to the clock time through the waiting-time PDF Equation (9). This means that we are dealing with a complex system undergoing a process of self-organization that generates crucial events. The number of degrees-of-freedom of this global system is so large that we observe only a small portion of it. This small portion interacts with its reservoir by quantum mechanical means. For this reason, we move from ρ(x,ξ,z,t) to p(x,t) and again turn the structure of Equation (50) into that of Equation (51). Applying this rule we obtain the generalized FDE:

(66) $$\frac{\partial }{\partial t}p\left(x,t\right)=D\int_{0}^{t}dt^{\prime }\Phi \left(t-t^{\prime }\right)t^{\prime 2H-1}\frac{\partial^{2}}{\partial x^{2}}p\left(x,t^{\prime }\right).$$

Comparing this equation to Equation (18) it is seen that the diffusion process described by Equation (66) is different from the FDE described by Equation (18). This is to be expected; Bologna et al. [22] observed first the quantum-like evolution of their system and then applied the Caputo fractional derivative. We have changed that order of operations to obtain Equation (66).

## 4. Equivalence of the Two Diffusion Equations

In spite of its different structure, Equation (66) is completely equivalent to Equation (18). Taking the Laplace transform of Equation (66) and using its convolution properties, we have:

(67) $$u\hat{p}\left(x,t\right)-p_{0}\left(x\right)=D\hat{\Phi }\left(u\right)L\left\{t^{2H-1}\frac{\partial^{2}}{\partial x^{2}}p\left(x,t\right);u\right\},$$

where $\hat{p}\left(x,u\right)$ and $\hat{\Phi }\left(u\right)$ are the Laplace transform of the PDF p(x,t) and the kernel Φ(t), respectively. Finally $L\left\{\cdot ;u\right\}$ stands for the Laplace transform of its argument. Using Equation (15), we obtain:

(68) $$u^{\alpha }\hat{p}\left(x,u\right)-u^{\alpha -1}p_{0}\left(x\right)=DL\left\{t^{2H-1}\frac{\partial^{2}}{\partial x^{2}}p\left(x,t\right);u\right\}.$$

The left side of Equation (68) is the Laplace transform of the Caputo fractional derivative, of order 0<α≤1, with respect to time, of p(x,t). Taking the inverse Laplace transform of this last equation yields:

(69) $$\frac{\partial^{\alpha }}{\partial t^{\alpha }}p\left(x,t\right)=Dt^{2H-1}\frac{\partial^{2}}{\partial x^{2}}p\left(x,t\right),$$

where the fractional time derivative is of Caputo form. Thus, we recover Equation (18) whose solution was determined by Bologna et al. [22]. This analysis shows that Equation (66) is completely equivalent to Equation (18) and, as a consequence, shares the same scaling prescription as that given by Equation (19).

## 5. Analysis of Time Series

The result of Section 4 simplifies the synthesis of surrogate sequences corresponding to Equation (18), which had been derived in [22] using phenomenological arguments. To generate such a sequence yielding the equivalent Equation (66), following the directions of Section 3, we generate a sequence of crucial times t(n), where t(n) locates the quantum mechanical values corresponding to $\left\{\xi ,z\right\}$. This corresponds to implementing the prescription of Equation (63) defining the times at which those values of the reservoir variables are located. We make the assumption that the projection approach illustrated in Section 3 turns the quantum mechanical variable $\hat{\xi }$ into one of the values of the sequence {ξ(t)} generating the autocorrelation function $\Phi_{\xi }\left(t\right)$.

We first implement the fBm [10] in the operational time *n* to generate ξ(n). These values are then assigned to the clock time t(n). The resulting time series is filled with many vanishing values and with the values ξ(n) occurring at the times t(n), which is the same procedure as that described in Section 1.2 to realize the CTRW prescription of Equation (10). A substantial difference is that the CTRW events are totally uncorrelated in the operational time, while here they express quantum coherence, as described earlier. We convert the time series into the diffusional trajectory $X\left(t\right)=\int_{0}^{t}dt^{\prime }\xi \left(t^{\prime }\right)$ and attempt to establish the roles of crucial events and quantum coherence by assessing the departure of the scaling of the diffusing trajectory X(t) from ordinary diffusion.

To make the results of our procedure useful for the analysis of signals generated by cognitive processes, we make our statistical evaluation using a single time series. We analyze the single diffusion process X(t) using a method of mobile windows as originally done in the pioneering work of Peng et al. [40]. We move the window of size *l* along the trajectory X(t) and the quantity Y(l,t)=X(t+l)−X(t) is calculated for each window position. This converts the single trajectory X(t) into many trajectories Y(l,t). The parameter *t* labels a random walker that at l=0 is located at the origin Y=0, in line with the comment made in Section 2 that diffusion processes are described by conditional probabilities.

Evaluating of the second moment to establish the nature of the departure from ordinary diffusion of the *Y*-diffusion process may lead to misleading results because crucial events generate Lévy diffusion with diverging second moments [37]. This problem was solved by the authors of [41] who proposed the method of diffusion entropy analysis (DEA) based on using the Shannon entropy to analyze the empirical PDF p(y,l) according to:

(70) $$S\left(l\right)=-\int_{-\infty }^{+\infty }dyp\left(y,l\right)ln\left[p(y,l)\right].$$

When p(y,l) obeys the scaling prescription of [41], DEA predicts an entropy of the form:

(71) $$S\left(l\right)=A+\delta ln\left[l\right],$$

where *A* is a constant and δ is the scaling index of the diffusion process. In the case where the source of anomalous diffusion is fBm, δ=H. However, this method does not detect the possible contribution of crucial events to anomalous diffusion if their scaling δ<H. Furthermore, if there are crucial events in action with μ<2, the non-stationary nature of the time series makes DEA inaccurate.

A significant improvement to the method was made by [42] focusing on the case where all the events of the time series under study are crucial. In this case the random walker makes a step ahead of constant length, for instance 1, and again the trajectory X(t) is converted into a set of diffusional trajectories Y(l,t) to obtain the scaling index:

(72) δ=μ−1,

for 1<μ<2, and the alternative form:

(73) $$\delta =\frac{1}{\mu -1},$$

for 2<μ<3.

To assess the possible role of quantum coherence we adopt a further augmented DEA supplemented by the use of stripes as discussed in [26] and referred to as Modified DEA (MDEA).

**Figure 1:**
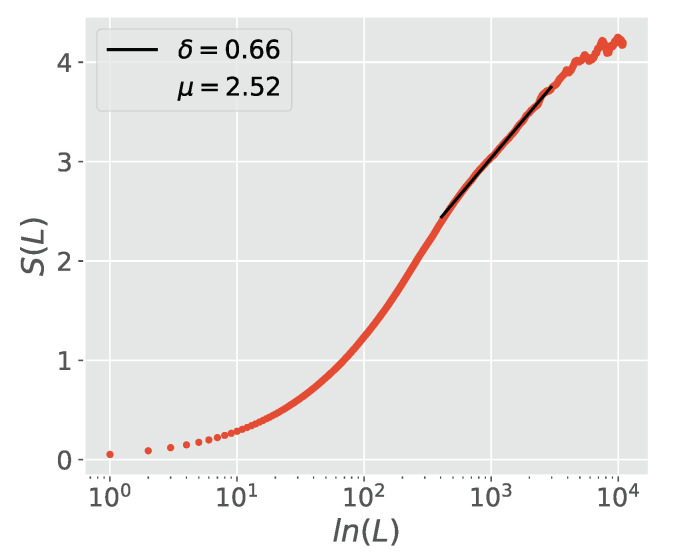
The entropy of the numerical time series ξ of pure crucial events, generated with an inverse power law (IPL) index μ=2.5. Calculated using MDEA with stripes of width 1 and plotted against the log of the window size *l*. The fluctuation ξ was generated by filling the laminar region between two crucial events with 1 or −1 decided by a fair coin toss. The dashed line corresponds to the fit with δ=0.66 which is essentially identical to Equation (73), that being, $\delta =\frac{2}{3}$.

Figure 1 shows the result of applying MDEA to a numerically generated time series when the IPL index for the time interval between crucial events is μ=2.5. This is a case where in the long time limit the process becomes ergodic, and methods less efficient than MDEA, such as the scaling of the second moment, also work. In this case the scaling δ is related to that for the crucial events time intervals μ by Equation (73) [42]. MDEA successfully resolves the scaling index given by Equation (73).

**Figure 2:**
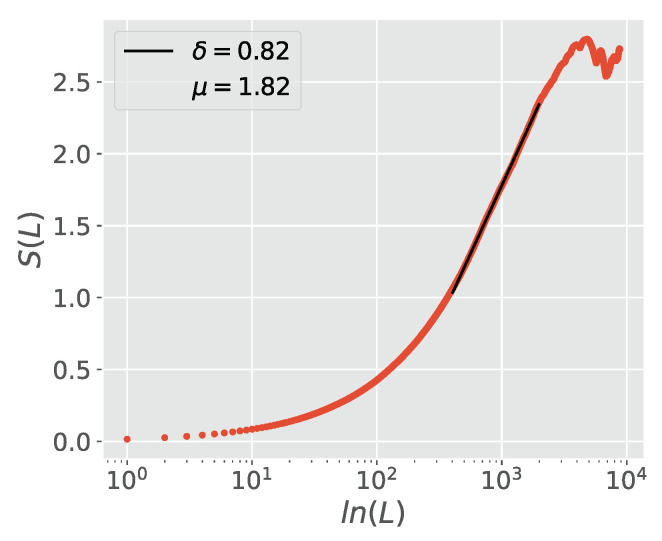
DEA performed with stripes of size s=1 on a sequence of pure crucial events, generated with an IPL index μ=1.8, with ξ generated by the same coin tossing prescription as in Figure 1.

Figure 2 shows that MDEA also works in the case where μ<2 where both the first and second moments of the time series diverge. This case corresponds to the theory developed in this article. Since the Caputo fractional derivative index is related to crucial event IPL index μ=1+α<2, Equation (72) predicts that δ=α, which is borne out in the figure. MDEA yields the same accurate results as the direct observation of crucial events as in [41].

**Figure 3:**
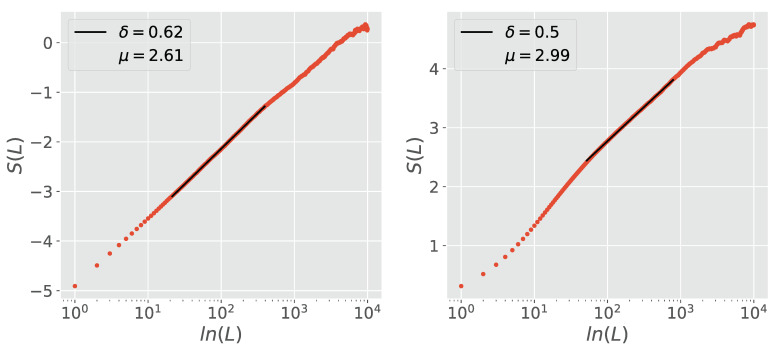
Comparison of DEA performed without (**left panel**) and with (**right panel**) stripes on a sequence of pure fractional Brownian motion (fBm), synthesized using H=0.6. The DEA without stripes yields a scaling δ equal to the *H* of the fBm, while the DEA with the stripes filters out the fBm memory and yields a scaling of δ=0.5.

**Figure 4:**
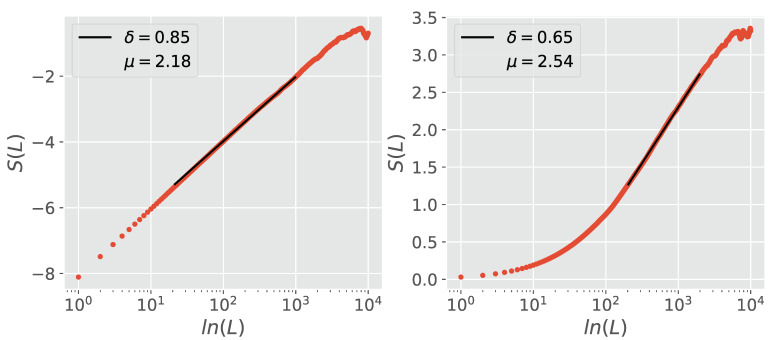
Comparison of DEA performed without (**left panel**) and with (**right panel**) stripes on a combined sequence of both fBm, generated using H=0.9, and crucial events generated using μ=2.5. The DEA with stripes filters the fBm memory and yields an δ much closer to that given by the true value of μ than the DEA without.

The use of only two stripes, one with ξ>0 and one with ξ<0, is sufficient to filter out the memory associated with fBm. The use of multiple stripes however, yields more accurate results as depicted in Figure 3 and Figure 4. These refer to the case where H≠0.5. Figure 3 refers to the case where δ>H, and shows that without the use of stripes DEA yields δ=H. MDEA filters the quantum coherence induced fBm and resolves the crucial scaling. Figure 4 shows that when δ<H, DEA without the use of stripes yields *H* as the scaling, while with stripes it yields the crucial scaling δ.

## 6. Concluding Remarks

### 6.1. Remarks on the Results Obtained

This article has been devoted to the joint action of fBm and the Caputo fractional derivative, interpreted as the joint action of quantum-like coherence and crucial events. Although the quantum derivation of fBm rests on quantum formalism [23,24], these results are limited to linear potentials whereas the Liouville equation of Wigner [27] is indistinguishable from the classical Liouville equation [28]. Advocates of QPT adopt a theoretical perspective based on the quantum mechanics of nonlinear systems. Our quantum mechanical treatment, being confined to linear potentials, is not sufficient to address bounded rationality. However, the analysis of real time series that we propose in this article may also apply to the case where the dynamical properties of the system under study are generated by nonlinear potentials. The work of [28] studies the dynamical process generated by the standard map for special values of a control parameter, which generates crucial events with a waiting-time distribution density identical to Equation (9). The same problem reveals inadequacies of the correspondence principle, because in the long-time regime a new physical condition is generated where the crucial events combine with the quantum mechanical constraints generated by wave functions spreading in the complex phase space of the anomalous standard map. In the classical case the ballistic islands, generating crucial events, are separated by a chaotic sea where the free diffusion of quantum trajectories is stopped upon time increase.

Another example of the dynamics generated by nonlinear potentials is found in [43]. In this case the long-time regime is characterized by quantum tunneling from one regular island to another. This phenomenon, called chaos-assisted tunneling, is of interest for quantum simulations [44] and our article may raise further interest for interpreting this as the joint action between crucial events and quantum-like coherence.

The recent paper [45] shows that the spectrum generated by heartbeats of autonomic neuropathy patients exhibits 1/f noise and MDEA shows that the parameter μ increases from the healthy condition, μ=2, towards the border of ordinary statistical physics, μ=3, as illness progresses. This is possible only if the heartbeats constitute a combination of fBm and crucial events, with fBm coming to dominate as illness progresses. Further research is required to determine the theoretical foundation of this transition process from crucial events dominated dynamics to quantum-like coherence dominated dynamics.

### 6.2. Implications for Future Research

Further research must be done to confirm these results and to strengthen the foundations for the rigorous theory, which may overlap with research on chaos-assisted tunneling. The Caputo fractional derivatives are manifestations of crucial events, a recent example of the relevance of which to complexity in network dynamics is found in [25]. In it, Turalska and West studied a social network of interacting individuals at criticality and found that the global complex network generates crucial events. The behaviors of single units in isolation would be to vacillate randomly between states; however, when the degree of units’ social attention is sufficiently intense, the behavior of the social system becomes critical. Then the behaviors of single units are described by Equation (14) and their change of opinion is described by a Mittag–Leffler survival probability [46]. This observation is compatible with the new research directions established by Tversky [47] and Kahneman [48], who used their insights from psychology to establish the importance of non-rational behavior of individuals in the theory of decision making while engaged in economic tasks. Individuals make fast-thinking decisions, influenced only by the opinions of their nearest neighbors, in apparent conflict with the assumed rationality in standard economic theory. For this reason we believe that the lack of commutation, P(A|B)≠P(B|A), pointed out by Yearsley and Busemeyer [6] is connected to the action of crucial events.

Further support for the proposition that bounded rationality properties of complex systems may be generated by criticality with no need to invoke quantum probability is lent by the the close connection between crucial events [13] and weak chaos [17]. The study [13] used an idealized version of the Manneville map, a popular approach to turbulence, to explain the occurrence of maximum ergodicity breakdown at μ=2. The study [17] observed that the condition 1<μ<3 is founded upon turbulence, with crucial evens interpreted as a short region of chaotic behavior separated by long time intervals of regular behavior, i.e., laminar regions. They defined the region 1<μ<3 as the weak chaos condition. This condition is now widely studied for physiological, sociological and ecological applications [49]. The recent work of Vandermeer [50] is of particular interest for its connection to this article. In it, Vandermeer illustrates a complexity condition corresponding to an Ising-like phase transition closely related to the criticality condition discussed in this paper, and argues it to be the main origin of bounded rationality.

In spite of the widely accepted conviction that the origin of bounded rationality requires quantum probability, we are inclined to believe that criticality is the source of the important discovery made by Tversky and Kahneman [5]. This manuscript affords the tools to support this conviction with the analysis of physiological data along the lines of the recent papers [7,45], showing that physiological processes are mixtures of coherence and crucial events. As shown by Tuladhar and co-workers [51], meditation has the effect of increasing heartbeat coherence, and coherence is a biological property that may be combined with criticality thanks to future theoretical developments. The work of [45] illustrating the progression of autonomic neuropathy on heartbeats, shows that crucial events are turned into ordinary Poisson events with the increase of disease severity, and the heartbeat’s dynamical complexity is turned into fBm coherence.

A theory explaining this interesting phenomenon does not yet exist, even if we do not rule out the possibility that the work done in quantum chaos in [43] may afford an exhaustive explanation involving the action of non-linear quantum mechanics on a dynamical process that in the classical condition would be characterized by weak chaos. The spreading of quantum wave functions in a phase space characterized by deterministic islands embedded in chaotic sea with a fractal border between regular islands and chaotic sea yields crucial events that in the long-time limit are turned in quasi-coherent tunneling. This manuscript affords the powerful tool of DEA illustrated in Section 5. This technique filtering out the anomalous effect of coherence will allow researchers to establish quantitatively the contributions of crucial events to the deviation from the ordinary scaling. Section 5 shows that the DEA with no stripes may generate a deviation from ordinary scaling larger than the DEA with stripes in the case where the process is a superposition of fBm and crucial events. This property is expected to be important to help the development of a theoretical approach explaining the joint action of fBm and crucial events.

## Data Availability

Data sharing not applicable.
